# Genome-wide analysis in *Hevea brasiliensis* laticifers revealed species-specific post-transcriptional regulations of several redox-related genes

**DOI:** 10.1038/s41598-019-42197-8

**Published:** 2019-04-05

**Authors:** Yi Zhang, Julie Leclercq, Shuangyang Wu, Enrique Ortega-Abboud, Stéphanie Pointet, Chaorong Tang, Songnian Hu, Pascal Montoro

**Affiliations:** 1 CIRAD, UMR AGAP, F-34398 Montpellier, France; 2 AGAP, Univ Montpellier, CIRAD, INRA, Montpellier SupAgro, Montpellier, France; 3 CAS Key Laboratory of Genome Sciences and Information, Beijing Institute of Genomics, Chinese Academy of Sciences, Beijing, 100101 China; 4 University of Chinese Academy of Sciences, Beijing, 100049 China; 5 Rubber Research Institute, Chinese Academy of Tropical Agricultural Sciences (CATAS), Danzhou, 571737 China

## Abstract

MicroRNA-mediated post-transcriptional regulation has been reported on ROS production and scavenging systems. Although microRNAs first appeared highly conserved among plant species, several aspects of biogenesis, function and evolution of microRNAs were shown to differ. High throughput transcriptome and degradome analyses enable to identify small RNAs and their mRNA targets. A non-photosynthetic tissue particularly prone to redox reactions, laticifers from *Hevea brasiliensis*, revealed species-specific post-transcriptional regulations. This paper sets out to identify the 407 genes of the thirty main redox-related gene families harboured by the *Hevea* genome. There are 161 redox-related genes expressed in latex. Thirteen of these redox-related genes were targeted by 11 microRNAs. To our knowledge, this is the first report on a mutation in the miR398 binding site of the cytosolic CuZnSOD. A working model was proposed for transcriptional and post-transcriptional regulation with respect to the predicted subcellular localization of deduced proteins.

## Introduction

Reactive oxygen species (ROS) are produced by redox reactions in plants, including aerobic respiration and photosynthesis. High levels of ROS such as ^1^O_2_ (singlet oxygen), O_2_°^−^ (superoxide radical), °OH (hydroxyl radical) and H_2_O_2_ (hydrogen peroxide) are generated during abiotic and biotic stress, as well as some plant development processes. This oxidative stress triggers disturbances in the basal redox state^1^. Peroxides and free radicals damage all cellular components including proteins, lipids and nucleic acids. ROS are also described as secondary messengers that are perceivable and able to initiate adaptive mechanism^2,3^. In order to detoxify the harmful ROS and maintain the redox homeostasis, plant cells contain enzymatic and non-enzymatic scavenging systems.

MicroRNA-mediated post-transcriptional regulation has been reported on ROS production and scavenging systems. This control can occur by transcript cleavage of either redox-related genes^4,5^, or their upstream transcription factors^6^, as well as indirectly through the repression of genes that induce hormone changes^7^ or a response to stress^8^. Although microRNAs first appeared highly conserved among plant species^9^, several aspects of biogenesis, function and evolution of microRNAs were shown to differ^10^. Non-conserved or species-specific microRNAs often expressed at very low levels could be detected using next-generation sequencing technology^11,12^. Besides microRNAs, little is known on the role of siRNAs on the expression of redox-related genes. Degradome analysis was first carried out in plant on Arabidopsis to facilitate the discovery and quantification of small RNAs cleaved targets^13^. Degradome sequencing experimentally confirmed several hundred targets in eucalyptus and populus^14,15^.

*Hevea brasiliensis* is the main commercial source of natural rubber, the *cis*-1,4-polyisoprene polymer, which is synthesized in the rubber particles of laticifers^16^. Latex is the cytoplasm of these articulated laticiferous vessels arranged in concentric rings in the phloem tissue. Latex flows out after cutting the soft bark (tapping). The application of ethephon, an ethylene releaser, to the bark stimulates latex flow and latex regeneration between two tappings^17^. ROS production takes place in laticifers in response to harvesting stress and consequent metabolic activity necessary for latex regeneration after tapping^18^. When ROS-scavenging systems cannot offset ROS accumulation, cellular dysfunctions lead to the agglutination of rubber particles^19,20^. This physiological syndrome, called Tapping Panel Dryness (TPD), is responsible for major losses in natural rubber production^21^.

Besides the evidence of ROS involvement in TPD at biochemical level^22^, several recent transcriptomic analyses reported that the expression of genes involved in the production and scavenging of ROS is regulated in latex. For instance, a comparison of two contrasting clones for latex yield showed that antioxidant-related genes are crucial in the regulation of latex regeneration and the duration of latex flow^23^. Juvenility was also found to be related to latex production. Latex from self-rooted juvenile clones created by somatic embryogenesis showed more differentially expressed genes (DEGs) related to the ROS-scavenging metabolism^24^. Transcriptomic analysis of a set of rubber clones showed that three and six overexpressed DEGs were involved in ROS production and ROS-scavenging, respectively^25^. Although all these genes were expressed in latex, several other studies did not report any significant changes in the expression of antioxidant genes in latex^26–28^. Post-transcriptional regulation by microRNAs was observed for some redox-related genes. Sixty-eight families of microRNAs, conserved between species, were identified in *Hevea*, including 15 with their precursors, and 16 species-specific microRNAs^11,29–33^. Approximately 1,000 targets were predicted and only a few targets have been experimentally validated to date^11,34^. All these studies globally analysed gene expression but did not specifically check redox-related gene families.

Laticifers are particularly prone to redox reactions. The latex of this non-photosynthetic tissue represents an interesting model to study how important are transcriptional and post-transcriptional regulations related to redox-related genes. This paper sets out to identify all the members of the most important gene families involved in the production and scavenging of ROS and their expression in latex, based on the new complete reference genome sequence^25^ and a transcriptome for a TPD-susceptible clone^26^. Of the 161 redox-related genes expressed in latex, 27 genes were shown to be targeted by microRNAs using small RNAs and degradome analyses. A working model was proposed for transcriptional and post-transcriptional regulations with respect to the predicted subcellular localization of deduced proteins. To our knowledge, this paper reports on the most complete classification of redox-related genes for a crop species, and reveals new insights into small RNA-mediated post-transcriptional regulations of ROS-scavenging systems.

## Results

### Identification and classification of redox-related genes in *Hevea*

**Figure 1:**
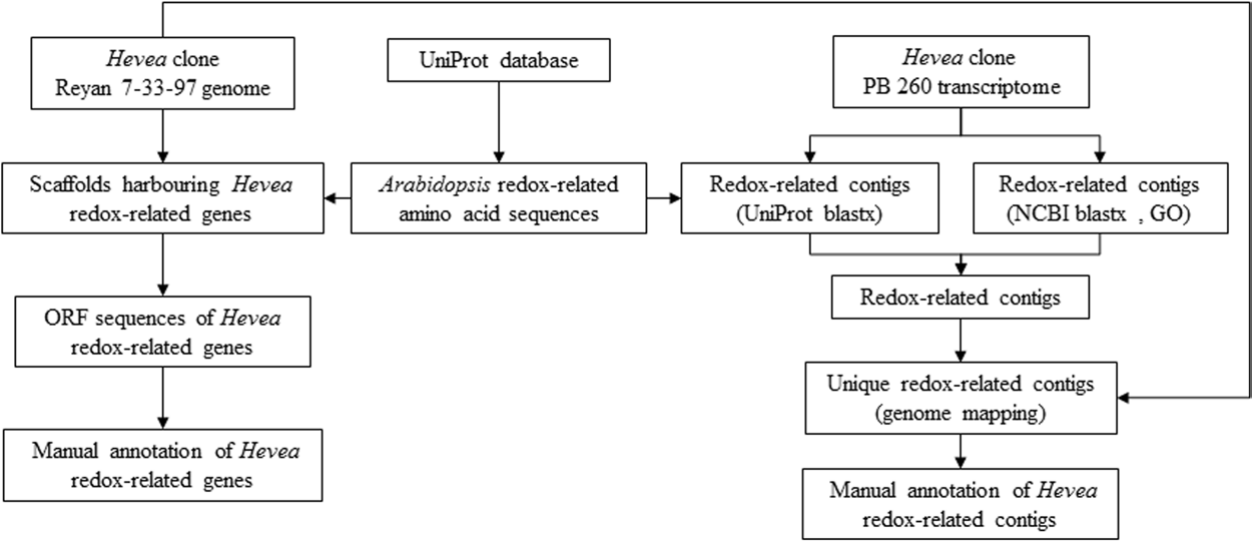
Workflow diagram illustrating the main steps in the identification of redox-related genes in the *Hevea* reference genome sequence and transcriptome. Reference redox-related amino acid sequences were downloaded from the UniProt database. These sequences were blasted against the *Hevea* genome and transcriptome. Scaffolds harbouring *Hevea* redox-related genes were validated manually with ORF. Redox-related contigs were also identified using blastx and GO annotations of the *Hevea* transcriptome. The two lists of contigs were merged and blasted against the *Hevea* genome to identify unique contigs. They were then manually annotated with ORF and genome mapping.

*Hevea* redox-related genes were identified in the rubber tree genome sequence from clone Reyan 7-33-97 using *Arabidopsis thaliana* or *Populus trichocarpa* amino acid sequences from 30 gene families downloaded from the UniProt database according to the procedure described in Fig. 1. *Hevea* genes were compared to eight other species based on a bibliographical analysis (Table 1). This analysis revealed that the redox-related gene families identified mostly dealt with ROS production and scavenging and partial information is available for antioxidant biosynthesis. The number of genes for each species was extracted from several references (Supplemental Table 1).

**Table 1:** Classification of main redox-related gene families in *Hevea brasiliensis* compared with several other species: *Arabidopsis thaliana, Manihot esculenta, Oryza sativa, Populus trichocarpa, Ricinus communis, Sorghum bicolor, Vitis vinifera, Zea mays*.

Function	Gene family	*Arabidopsis*	*Hevea*	*Manihot*	*Oryza*	*Populus*	*Ricinus*	*Sorghum*	*Vitis*	*Zea*
ROS production	Respiratory burst oxidase homolog	10	9	11	9	10	9	13	8	18
	Polyphenol oxidase	0	6	1	2	11	1	8	4	6
ROS scavenging and regulation	Peroxidase	73	114	—	138	87	—	140	—	—
	Catalase	3	5	10	3	4	2	2	6	3
	Superoxide dismutase	8	9	16	7	10	8	5	12	11
	Ascorbate peroxidase	7	8	19	11	11	10	8	8	16
	Glutathione peroxidase	8	10	7	6	7	5	6	5	5
	Glutathione reductase	2	3	5	3	3	3	2	2	2
	Monodehydroascorbate reductase	5	6	6	5	3	3	5	3	4
	Dehydroascorbate reductase	4	3	3	2	4	4	2	3	2
	Methionine sulfoxide reductase	14	9	—	7	9	—	—	—	6
	Peroxiredoxin	10	10	9	11	12	7	6	9	6
	NADPH-dependent thioredoxin reductase	3	3	—	3	3	—	3	2	—
	Glutathione S-transferase	51	77	—	84	81	—	99	—	72
	Glutaredoxin	43	51	—	49	38	—	32	25	—
	Thioredoxin	38	54	—	46	45	—	29	32	23
Ascorbate biosynthesis	GDP-L-galactose phosphorylase	2	3	—	1	2	—	—	2	1
	GDP- mannose pyrophosphorylase	3	2	—	3	—	—	—	1	—
	GDP-mannose-3′,5′ epimerase	1	2	—	2	2	—	—	2	—
	L-galactono-1,4-lactone dehydrogenase	1	1	—	2	1	—	—	1	1
	Inositol phosphate phosphatase	1	2	—	1	3	—	—	1	1
	L-galactose dehydrogenase	1	3	—	1	2	—	—	2	1
	L-gulonolactone oxidase	7	3	—	—	—	—	—	1	—
	Myo-inositol oxygenase	5	3	—	1	—	—	—	2	—
Glutathione biosynthesis	Glutamate cysteine ligase	1	2	—	1	2	—	—	—	1
	Glutathione synthetase	1	2	—	—	2	—	—	—	1
Tocotrienol biosynthesis	Tocotrienol cyclase	1	1	—	1	—	—	—	—	—
	Tocotrienol γ-methyltransferase	1	2	—	1	—	—	—	—	—
	MPBQ/MSBQ methyltransferase	1	3	—	1	—	—	—	—	—
	Homogentisate phytyltransferase	1	1	—	2	—	—	—	—	—
In total		306	407	>87	>403	>352	>52	>360	>131	>180

*Hevea* has a much larger number of redox-related genes (407) compared to *Arabidopsis* (306). This is mainly explained by the absence of genes encoding polyphenol oxidase in *Arabidopsis* when *Hevea* genome harboured 6 genes, and by a smaller number of genes encoding glutaredoxin (43), glutathione S-transferase (51) and peroxidase (73) in *Arabidopsis* compared to *Hevea* (51, 77 and 114, respectively). A phylogenetic analysis was carried for gene families involved in ROS production and scavenging (Supplemental Figs 1–17). This analysis revealed several gene duplications for Grx, GST and Px gene families (Supplemental Figs 7, 8 and 14).

### Comparative analysis of published latex transcriptomes

In order to identify redox-related genes expressed in latex, contigs or unigenes annotated as redox-related genes were extracted from the Supplemental Table 2 of recently published latex transcriptome analyses obtained by RNA sequencing technology^23–28^. For each publication, redox-related contigs or unigenes were assigned to one of the 30 gene families using their initial blastx annotation (Table 2). A small number of contigs (28, 30 and 12) was counted for three studies^23,24,28^ compared to the total gene number found in this work (Table 1) and other transcriptome analyses (912, 77, 231)^25–27^. The transcriptome published by Wei and collaborators had the largest number of redox-related contigs (234) but a lower coverage (0.37 Gb for all samples)^27^. This transcriptome was obtained from trees of rubber clone RRIM 600 with long-term latex flow. For several gene families, the number of contigs was larger than the gene number counted in the reference genome. Tang and co-workers published transcriptome data for a mixture of several tissues including latex. Thus, the RNAseq dataset from clone PB 260^26^ was adopted for further analysis for the following reasons: high coverage (6 Gb per sample), largest number of redox-related contigs (912), representation of all gene families, good statistical design with the use of 3 biological replicates, and data from a comparison of latex from healthy and TPD-affected trees.

**Table 2:** Annotation of *Hevea* latex redox-related genes from published latex transcriptomes.

**Reference**	Chao 2015	Li 2015	Wei 2015	Li 2016	Tang 2016	Montoro 2018	This study
**Topic**	Rubber yield	Rubber yield	Latex flow	Rubber yield	Genome	TPD	Redox
**Technology**	Hiseq2000	Hiseq2000	Hiseq2500	Hiseq2000	Hiseq2000	Hiseq2000	—
**Throughout**	35 Mb	4.82 Gb	0.37 Gb	16.7Mbp	1.29 Gb	6 Gb	—
**Clone**	CATAS8-79 PR107	RRIM 600 RY 7-20-59	RRIM 600	CATAS7-33-97 HAIKEN 2	Reyan7-33-97	PB 260	PB 260
**Tissue**	latex	latex	latex	latex	Mixed tissues	Latex	Latex
**Gene family**	**Number of contigs or unigenes**						
Respiratory burst oxidase homolog	1	1	4	0	1	26	2
Polyphenol oxidase	2	0	1	1	1	4	2
Peroxidase	6	2	18	2	5	145	7
Catalase	0	0	8	1	3	31	3
Superoxide dismutase	2	0	14	1	1	43	6
Ascorbate peroxidase	1	0	15	0	1	27	5
Glutathione peroxidase	0	1	10	0	1	45	7
Glutathione reductase	3	0	4	0	1	17	3
Monodehydroascorbate Reductase	1	1	5	0	2	26	5
Dehydroascorbate reductase	0	2	3	0	1	7	3
Methionine sulfoxide reductase	1	1	7	0	3	24	7
Peroxiredoxin	2	0	12	0	2	42	7
NADPH-dependent thioredoxin reductase	0	2	2	0	7	8	3
Glutathione S-transferase	5	6	44	3	16	93	23
Glutaredoxin	2	2	16	0	4	104	20
Thioredoxin	0	10	43	3	21	189	32
GDP-L-galactose phosphorylase	0	0	4	0	1	3	2
GDP- mannose pyrophosphorylase	0	0	0	0	0	3	2
GDP-mannose-3′,5′ epimerase	0	1	3	0	1	7	2
L-galactono-1,4-lactone dehydrogenase	0	0	3	0	0	7	1
Inositol phosphate phosphatase	0	0	0	0	0	7	2
L-galactose dehydrogenase	0	0	0	0	1	3	3
L-gulonolactone oxidase	0	0	2	0	0	16	1
Myo-inositol oxygenase	1	0	2	1	0	7	3
Glutamate cysteine ligase	1	0	2	0	0	5	2
Glutathione synthetase	0	0	1	0	0	4	2
Tocopherol cyclase	0	0	1	0	0	4	1
Tocopherol γ-methyltransferase	0	0	2	0	0	6	1
MPBQ/MSBQ methyltransferase	0	0	3	0	2	3	3
Homogentisate phytyltransferase	0	1	2	0	2	6	1
**Total contigs or unigenes**	28	30	231	12	77	912	161

### Transcriptional regulation of redox-related genes and prediction of subcellular localization in laticifers

Of the 407 *Hevea* redox-related genes, 161 unique transcripts were found in latex (Supplemental Table 2). All transcripts were encoded by a unique gene, except for 3 transcripts encoded by two genes harboured by 2 different scaffolds, respectively: CL1895Contig4 (L-galactose dehydrogenase 1 (GDH1) and L-galactose dehydrogenase 2 (GDH2); CL3344Contig2 (glutathione S-transferase U8; GSTU8) and glutathione S-transferase U11 (GSTU11); and CL2806Contig1 (NADPH-dependent thioredoxin reductase 1; NTR1) and NADPH-dependent thioredoxin reductase 3; NTR3). NTR1 and NTR3 were located on scaffold0536_346249 and scaffold0965_30248. GSTU8 and GSTU11 were located on scaffold0702_452766 and scaffold0702_454607. GDH1 and GDH2 were located on scaffold1364_78602 and scaffold1364_29743. The phylogenetic analyses revealed a recent duplication of the genes (Supplemental Figs 4, 8 and 11).

Subcellular localization of redox-related genes was performed using WoLF PSORT, CELLO2GO and Plant-mPLoc. The largest number of proteins was predicted in chloroplast. Given that laticifers are non-photosynthetic tissues, chloroplast and plastid predictions were assigned as plastidic proteins. Subcellular localization of latex proteins was predicted as follows: 82 in plastids, 70 in cytosol, 12 in nucleus, 7 in mitochondria, 2 in extracellular, 1 in vacuole, 2 in peroxisome and 7 non-predicted.

**Figure 2:**
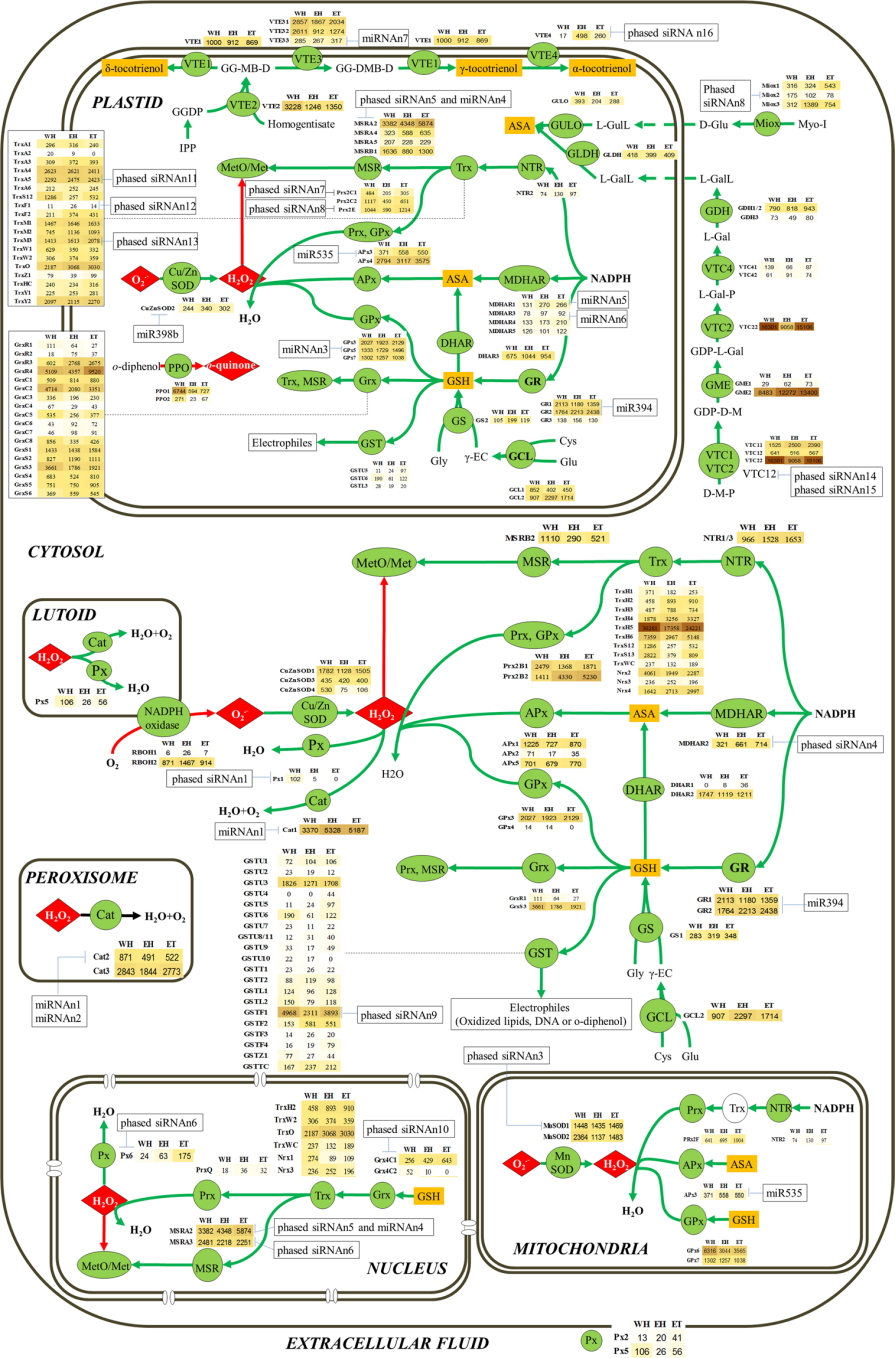
ROS production and scavenging systems, and antioxidant biosynthesis in the various latex cell compartments. The gene expression level is represented using RNAseq reads. The data in the three columns originate from heathy trees without ethephon treatment (WH), healthy trees with ethephon treatment (EH) and tapping panel dryness trees with ethephon treatment (ET), respectively. The red arrows represent ROS production or oxidation events. The green arrows represent ROS scavenging reactions or reduction events. Abbreviations are: superoxide radicals (O_2_^•−^), hydrogen peroxide (H_2_O_2_), catalase (Cat), peroxidase (Px), ascorbate peroxidase (APx), glutathione peroxidase (GPx), peroxiredoxin (Prx), glutathione (GSH), monodehydroascorbate reductase (MDHAR), dehydroascorbate reductase (DHAR), glutathione reductase (GR), glutamate cysteine ligase (GCL), glutathione synthetase (GS), γ-glutamylcysteine (γ-EC), L-glutamate (Glu), cysteine (Cys), glycine (Gly), NADPH reductase (NTR), thioredoxin (Trx), methionine sulfoxide (MetO), methionine sulfoxide reductase (MSR), glutaredoxin (Grx), glutathione S-transferase (GST), myo-inositol oxygenase (Miox), L-gulonolactone oxidase (GULO), myo-Inositol (Myo-I), D-Glucuronate (D-Glu), L-Gulono-1, 4-lactone (L-GulL), GDP-D-mannose pyrophosphorylase (VTC1), GDP-L-galactose phosphorylase (VTC2), D-Mannose-1-P (D-M-P), GDP-D-Mannose (GDP-D-M), GDP-mannose 3, 5-epimerase 1 (GME), GDP-D-M, GDP-L-Galactose (GDP-L-Gal), L-Galactose-1-P (L-Gal-P), inositol phosphate phosphatase (VTC4), L-Galactose (L-Gal), L-galactose dehydrogenase (GDH), L-Galactono-1, 4-lactone (L-GalL), L-galactono-1, 4-lactone dehydrogenase (GLDH), isopentenyl diphosphate (IPP), geranylgeranyl diphosphate (GGDP), homogentisate phytyltransferase (VTE2), 6-Geranylgeranyl-2-methylbenzene-1,4-diol (GG-MB-D), MPBQ/MSBQ methyltransferase (VTE3), 6-Geranylgeranyl-2, 3-dimethylbenzene-1, 4-diol (GG-DMB-D), tocopherol cyclase (VTE1), tocopherol γ-methyltransferase (VTE4), glutamate cysteine ligase (GCL).

When exploring RNAseq data from latex^26^, sixty transcripts were abundant (>1000 reads), and twelve of them were very abundant (>5000 reads) for one or other of the conditions. Twenty-nine transcripts were induced and forty-eight repressed in response to ethephon in healthy trees. Nine transcripts were induced in response to ethephon in TPD-affected trees. Four of these genes (PPO2, PrxQ, TrxS12 and TrxS13) showed contrasting regulation: repressed in healthy and overexpressed in TPD-affected trees. For the clarity of this manuscript, gene expression data are presented in Fig. 2 (cf. discussion section).

### Small RNA-mediated post-transcriptional regulation of redox-related genes

Redox-related transcripts targeted by microRNAs and ta-siRNAs were searched using CLEAVELAND pipeline^13^ in the degradome dataset obtained from various tissues (root, leaf, bark, latex, flowers and embryo) and the reference transcriptome for rubber tree clone PB 260. The degradome analysis did not revealed post-transcriptional regulations by ta-siRNA (data not shown). Of the 407 redox-related genes, 13 were targeted by 11 different microRNAs (Table 3). The degradome analysis revealed post-transcriptional regulation of these transcripts at spatial level (thanks to tissue-specific libraries) and cleaved transcript abundance level classified into degradome categories. The number of microRNA families was different for each tissue: 1 in roots, 7 in leaves, 7 in latex, 4 in bark and 2 in flowers. In latex, seven of these redox-related genes were targeted by 6 families of microRNAs. Three known families of microRNA (miR535, miR398b and miR394) targeted and cleaved transcripts from genes *APX3* (*ascorbate peroxidase 3*), *SOD*2 (*Cu/Zn superoxide dismutase* 2), *GR1* and *GR2* (*glutathione reductase 1* and *2*), respectively. For transcripts from gene *APX3*, strong spatial regulation was observed with a greater abundance of miR535 in leaf (degradome category 0) compared to latex (degradome category 4). One transcript, encoding MPBQ/MSBQ methyltransferase 3 involved in tocotrienol biosynthesis, was also cleaved in bark and leaf by a new microRNA named miRNAn7. For miR398b, which cleaves chloroplastic Cu/Zn superoxide dismutase transcripts^29^, a low abundance of cleaved transcripts was found in latex (degradome category 3) and root (degradome category 4). Interestingly, the three cytosolic isoforms were not detected in the degradome libraries confirming the previous observation made by Gébelin and co-workers^11^. The miR398 binding site was further scanned and sequence variations were observed in the 5′ and 3′ seed regions but also between the very sensitive 10^th^ and 11^th^ nucleotides of the microRNA sequence targeting *HbCuZnSOD1* and at the 12^th^ nucleotide in both *HbCuZnSOD3* and *HbCuZnSOD4* sequences (Table 4). Three microRNAs (miRNAn1 to 3), with cleavage activity in latex, were new microRNAs not yet annotated in the miRBase database (Table 3). *Catalase 1*, the unique cytoplasmic *Cat* gene showing the highest *Cat* expression in latex, was regulated by a new microRNA named miRNAn1. This mRNAn1 also targeted peroxisomal *catalase 2*. *Cytosolic glutathione reductase 1* and *plastidic glutathione reductase 2* were highly expressed in latex and targeted by miR394.

**Table 3:** Degradome data analysis with CLEAVELAND pipeline using 161 ROS-related genes, 6 tissue-specific transcriptomes and newly annotated microRNAs.

Target	Degradome			MicroRNA				
Enzyme	Contig	Library	Category	miRNA accession	miRBase annotation	Start position	Stop position	Cleavage site
Ascorbate peroxidase 3	CL1Contig1117	leaf	0	Pmature12390	miR535	53	73	64
		latex	4	Pmature12390	miR535	53	73	64
Catalase 1	CL1Contig10534	latex	3	Pyoung21016	miRNAn1, in progress	588	608	599
Catalase 2	CL1Contig1382	latex	3	Pyoung21016	miRNAn1, in progress	421	441	432
		latex	4	Pyoung160064	miRNAn2, in progress	422	442	433
Cu/Zn superoxide dismutase 2	CL1553Contig1	root	4	acc_420	miR398b	630	656	646
		latex	3	acc_420	miR398b	630	656	646
Glutathione peroxidase 5	CL449Contig1	leaf	0	Pmature37668	miRNAn3, in progress	70	90	81
		latex	4	Pmature37668	miRNAn3, in progress	70	90	81
Glutathione reductase 1	CL1Contig15684	bark	4	Pyoung83898	miR394	477	500	488
		leaf	2	Pyoung83898	miR394	477	500	488
		latex	2	Pyoung83898	miR394	477	500	488
Glutathione reductase 2	CL1Contig1556	leaf	2	Pyoung83898	miR394	560	583	571
		latex	2	Pyoung83898	miR394	560	583	571
Methionine sulfoxide reductase A2	CL372Contig4	bark	2	health2164	miRNAn4, in progress	210	231	222
		leaf	3	health2164	miRNAn4, in progress	210	231	222
Monodehydroascorbate reductase 1	CL1Contig7966	bark	2	Pmature18863	miRNAn5, in progress	149	170	161
Monodehydroascorbate reductase 3	CL1250Contig6	bark	2	Pyoung84691	miRNAn6, in progress	1181	1203	1194
MPBQ/MSBQ methyltransferase 3	CL5665Contig1	leaf	4	Pyoung169157	miRNAn7, in progress	951	973	962
		flower	2	Pyoung169157	miRNAn7, in progress	951	973	962
Myo-inositol oxygenase 2	CL234Contig10	flower	2	Pyoung68471	miRNAn8, in progress	401	424	415
Peroxidase 6	CL1Contig8355	leaf	2	Pyoung84691	miRNAn6, in progress	970	990	982

**Table 4:** Comparison of HbmiR398 (acc_420) cleavage site between cytosolic and chloroplastic CuZnSOD isoforms.

Gene name	Sub-cellular localization	mfe kcal/mol	Alignment
*HbCuZnSOD1*	cytosolic	Non functional	
*HbCuZnSOD2*	chloroplastic	−37.3	
*HbCuZnSOD3*	cytosolic	Non functional	
*HbCuZnSOD4*	cytosolic	Non functional	

Arrow indicated the cleavage site observed experimentally for *HbCuZnSOD2* by miR398 (Gébelin *et al*. 2012) and in the degradome analysis. Sequence variations in cytosolic isoforms sequences compared to *HbCuZnSOD2* are in bold and highlighted character.

The expression of the 13 post-transcriptionally regulated genes was recalculated using the reads covering the cleavage site only, in order to check if the level of expression assessed by the number of reads describes the real functionality of mRNAs (Supplemental Tables 3 and 4). The expression of 8 of the 13 targeted transcripts were significantly affected by the new way of calculation. Significant fold changes observed in standard RNA sequencing for ethephon treatment or TPD occurrence disappeared for genes *APX3, GR1, MDHAR2*, *Prx2C1, Px1, Px6* and *VTE4* when using the number of reads covering the cleavage site to calculate the expression level. Finally, some effects of ethephon were maintained for *GSTF1* and *Prx2C1*.

## Discussion

Apart from plant model species, this study is the most complete genome-wide analysis of ROS production and scavenging systems and antioxidant biosynthesis in a perennial crop. The main 30 redox-related gene families totalize 407 genes in *Hevea*. This is a larger number of genes compared to *Arabidopsis* especially due to the expansion of peroxidase genes in *Hevea*. Based on the RNAseq dataset, small RNA/target identification, and prediction of subcellular localization, a model of transcriptional and post-transcriptional regulations of the 161 redox-related genes expressed in latex was attempted for a rubber clone particularly prone to oxidative stress (Fig. 2). The redox-related proteins were predominantly localized in plastids (82 proteins) and cytosol (70 proteins). This comprehensive analysis highlighted critical steps of redox homeostasis in latex.

This study also revealed specific regulation of ROS-scavenging systems, which might be adapted to strong and steady ROS production in latex cells due to recurrent harvesting stress and latex regeneration between two tappings. Lutoids are polydispersed vacuoles with lysosomal properties. Previous biochemical studies revealed that NADPH oxidase is the main source of ROS in laticifer cytosol, especially under stress^18^. The present study revealed that this enzyme was mostly encoded by the *Rboh2* gene in latex cells, and its expression was enhanced by ethephon application. These results suggest that *Rboh2* encodes the main enzyme generating ROS at the outer surface of lutoid membrane in contact with cytosol.

This production of O_2_^−^ in cytosol requires a powerful detoxification system in this compartment. Superoxide dismutase is the enzyme involved in the first step of detoxification inducing the dismutation of the superoxide anions, produced by the lutoid NADPH oxidase, into hydrogen peroxide^22^. *CuZnSOD1* transcripts were much more abundant compared to other genes encoding SOD. Unlike *Arabidopsis*, none of the *Hevea* cytosolic SOD isoforms was subjected to post-transcriptional regulation by miR398. A mutation in the binding site makes miR398 ineffective. The high expression of the *CuZnSOD1* gene might then support the maintenance of SOD activity and a consequent high level of anion superoxide dismutation. To demonstrate the biological relevance of post-transcriptional regulations, the physiological context (type and duration of stress) in which the regulation occurs should be further identified case by case. For example, the cleavage of the *chloroplastic CuZnSOD* transcripts was correlated with the upregulation of miRNA398 expression in response to a salt stress specifically in bark and root^29^.

The second step deals with the decomposition of H_2_O_2_ to H_2_O and O_2_ through five hydrogen peroxide scavenging pathways coexisting in cytosol (peroxidase, ascorbate peroxidase, glutathione peroxidase, peroxiredoxin and catalase). High and steady ROS production in latex cells requires Cat activity, which generally comes into play under stress. A decrease in Cat activity was recorded in TPD-affected trees enabling the general oxidative stress in latex cells^35^. *Cat1* gene was highly expressed in latex and might be the main gene related to the Cat activity. Although post-transcriptional regulation was shown by microRNA miRn1, this microRNA did not efficiently cleave *Cat1* transcripts in the tested biological conditions (low number of read ends at the cleavage site in degradome data). For the genes encoding thioredoxins, *TrxH5* had the highest level of expression out of the 161 genes expressed in latex. From our knowledge, there is no published information related to the potential role of Prx in latex and further characterization is required. The ascorbate/glutathione cycle, involving in its last lines APx and GPx, is essential in the reduction of H_2_O_2_ to H_2_O and O_2_. Regeneration of the ASA and GSH forms reduced by the ascorbate-glutathione cycle involved several enzymes encoded by *MDHAR2*, *DHAR2*, *GR1* and *GR2*. The ethephon treatment did not transcriptionally activate genes involved in the glutathione/ascorbate cycle. Although some post-transcriptional regulations appeared in the degradome analysis showing that both the *GR1* and *GR2* transcripts, miR394 did not significantly cleave GR transcripts. APx has a high affinity for H_2_O_2_ and can reduce it to H_2_O in chloroplasts, cytosol, mitochondria and peroxisomes, as well as in the apoplastic space. Of the three genes encoding a cytoplasmic ascorbate peroxidase, the *HbAPx1* and *HbAPx5* transcripts were the most abundant. Considering the lower expression of these 3 *APx* genes compared to the plastidic *APx4*, the cytosolic ASA pathway might have a lower reducing capacity than the plastid pathway, which is obvious since the production of ROS is known to be high in plastids. Of the 23 *Hevea* genes encoding a GST, 21 were predicted as cytosolic GST. Among them, the *GSTU3* and *GSTF1* genes were actively expressed in latex cells. As GST plays a central role in the use of the reduction power of GSH to detoxify electrophiles, glutathione might be considered as the most important antioxidant in laticifers.

Glutathione, ascorbate and vitamin E isomers are the major antioxidants in latex^22^. The glutathione biosynthesis pathway involves two ATP-dependent enzymes: γ–glutamate cysteine ligase (GCL) and glutathione synthetase (GS). Of the two GS and GCL genes identified in the rubber genome, only one of each was encoded protein predicted to be expressed in latex cytosol (GS1 and GCL2), one GS (GS2) and the two GCL (GCL1 and GCL2) being expressed in plastids. The genes encoding GS2 and GCL2 were significantly over-expressed in response to ethephon. There are four routes for ASA biosynthesis in plant: the L-galactose pathway, the *myo*-inositol oxygenase pathway, the salvage pathway via L-galactonate, and the L-gulose-pathway. Of these four routes, L-galactose is the major pathway in many plants^36,37^. The L-galactono-1 4-lactone (L-GalL) biosynthesis pathway occurs in cytosol, which consists of five enzymes (VTC1, GME, VTC2, VTC4 and GDH). All genes encoding these enzymes have homologues expressed in latex cytosol.

There are 4 vitamin E isomers in latex: α-tocopherol, α-tocotrienol, γ-tocotrienol and δ-tocotrienol^38,39^. Genes involved in the biosynthesis of δ-tocotrienol (*VTE1*and *VTE2*) and γ-tocotrienol (*VTE1, VTE31*, *VTE32* and VTE33) were expressed at moderate or high levels in latex. VTE33 had also a low level of expression related to its targeting by miRNAn7. As γ-tocotrienol is the most abundant vitamin E isomer, its accumulation might be fostered by the weak capacity to produce α-tocotrienol and tocopherol.

To conclude, this study reveals new insights into small RNA-mediated post-transcriptional regulations of ROS-scavenging systems. To our knowledge, this is the first report on a mutation in the miR398 binding site of the CuZnSOD altering the post-transcriptional regulation described in model species. In addition, the literature mentioned microRNA-mediated post-transcriptional regulation on ROS production and scavenging systems. This work paves the way to the study of adaptive mechanisms. Besides, several genetic studies have revealed the involvement of antioxidant compounds in complex traits of several species^40–43^. In *Hevea*, the 161 redox-related genes expressed in latex represent candidate genes for the identification of allelic variability. The development of molecular markers and the analysis of genetic variability of antioxidants should support breeding programmes, especially for traits relative to environmental stress.

## Methods

### Identification and classification of redox-related genes in the *Hevea brasiliensis* genome and transcriptome

Redox-related genes were identified from both the *Hevea* reference genome and transcriptome (Fig. 1). An amino acid sequence dataset was created by downloading sequences of thirty redox-related gene families from the UniProt database (http://www.uniprot.org/) using published accession numbers mostly from *Arabidopsis*, except for the polyphenol oxidase (PPO) family, which is absent in *Arabidopsis*. Sixteen families were selected for ROS production and scavenging (Table 1). In addition, protein sequences of genes involved in the biosynthesis of three major antioxidants in latex (ascorbate, glutathione, and tocotrienol) were collected. This dataset was blasted against the published *Hevea* genome^25^ and transcriptome^26^. Redox-related contigs were also identified using blastx and GO annotations of the *Hevea* transcriptome. The two lists of contigs were merged and blasted on the rubber genome to identify unique contigs. Redox-associated genes were classified for each gene family related to ROS production, ROS-scavenging and regulation, and antioxidant biosynthesis (ascorbate, glutathione and tocotrienols).

### Phylogenetic analysis of redox-related genes

The full length amino acid sequences of *Arabidopsis* redox-related protein were aligned with the amino acid deduced sequences from *Hevea* clone Reyan 7-33-97 genome. Identities of proteins are provided in Supplemental Table 5. The polyphenol oxidase family being absent in *Arabidopsis*, we used the *Populus* PPO gene family. This alignment was made by Muscle via Mega 6^44^. Amino acid sequence of Arabidopsis actin 1 or Arabidopsis glutamate cysteine ligase was used as outgroup control. The phylogenetic trees were generated in Mega 6 by Bootstrap method with 500 replications after alignment.

### Prediction of the subcellular localization of redox-related proteins

The subcellular location of redox-related genes was predicted with translated sequences using WoLF PSORT (http://www.genscript.com/wolf-psort.html), CELLO2GO (http://cello.life.nctu.edu.tw/cello2go/) and Plant-mPLoc (http://www.csbio.sjtu.edu.cn/bioinf/plant-multi/). The 3 predictors were successfully tested on subcellular localization prediction^45^. The matching ratio between the prediction result and protein location was calculated according to Xiong’s Supplemental Table 2. The matching ratios from these 3 predictors ranged from 50% to 80%. The prediction of subcellular localization was considered as acceptable when the matching ratio of merged results was above 90%.

### Identification of small RNA and target mRNA couples

Degradome data for several *Hevea* tissues (latex, leaves, male and female flowers, seeds, root, bark and somatic and zygotic embryos) were obtained according to a protocol adapted from German^46^. *Hevea* microRNAs from small RNAseq data published by Gébelin and co-workers^11,29^ were annotated by MITP (https://sourceforge.net/projects/mitp/files/). This pipeline complies to the recommendations set by Axtell and coll^47^, looking from hairpin structures, producing miRNA and miRNA* with up to 3 bulges or 6 unpaired bases between miRNA and miRNA*. The prediction was done with sequences of 20–22 nt in size from 5 distinct small-RNA-seq libraries as recommended and not based on prediction from genomic sequences only. Degradome data were then analysed using the CLEAVELAND pipeline developed by Addo-Quaye^13^. The degradome categories correspond to: category 4: just one read at this position; Category 3: > 1 read, but below or equal to the average depth of coverage on the transcript; Category 2: > 1 read, equal to the average depth of coverage on the transcript; Category 1: > 1 read, equal to the maximum of the average depth of coverage on the transcript when there is >1 position at maximum value; Category 0: > 1 read, equal to the maximum of the average depth of coverage on the transcript when there is just one position at maximum value.

### RNA-seq data mining of cleaved targets

Expression of cleaved transcripts related to redox genes were calculated from the same RNA-seq datasets, with the exact number of reads overlapping the sRNA binding site, by using BEDTOOLS program (2.24.0) to intersect bam files with sRNA binding site coordinates (between Tstart and Tstop) provided by CLEAVELAND outputs. Then, by using R package EdgeR, comprising an over-dispersed Poisson model taking into account both biological and technical variability, differential gene expression analyses of replicated count data were performed^26^. The experimental design allows side-by-side comparison to identify firstly, differentially expressed genes upon ethephon stimulation in the latex of healthy trees, and secondly, differentially expressed genes in the latex of healthy and TPD-affected trees subjected to ethephon stimulation.

## Supplementary information

Supplementary figures

Supplementary Tables

## References

[1] Karkonen A, Kuchitsu K (2015). Reactive oxygen species in cell wall metabolism and development in plants. Phytochemistry.

[2] Baxter A, Mittler R, Suzuki N (2014). ROS as key players in plant stress signalling. J Exp Bot.

[3] Foyer CH, Noctor G (2005). Oxidant and antioxidant signalling in plants: a re-evaluation of the concept of oxidative stress in a physiological context. Plant, Cell & Environment.

[4] Guan Q, Lu X, Zeng H, Zhang Y, Zhu J (2013). Heat stress induction of miR398 triggers a regulatory loop that is critical for thermotolerance in Arabidopsis. Plant J.

[5] Naya L (2014). Regulation of copper homeostasis and biotic interactions by microRNA 398b in common bean. PLoS One.

[6] Yue E (2017). Overexpression of miR529a confers enhanced resistance to oxidative stress in rice (Oryza sativa L.). Plant Cell Rep.

[7] Zhang X, Wang W, Wang M, Zhang HY, Liu JH (2016). The miR396b of Poncirus trifoliata Functions in Cold Tolerance by Regulating ACC Oxidase Gene Expression and Modulating Ethylene-Polyamine Homeostasis. Plant Cell Physiol.

[8] Yuan S (2015). Constitutive Expression of Rice MicroRNA528 Alters Plant Development and Enhances Tolerance to Salinity Stress and Nitrogen Starvation in Creeping Bentgrass. Plant Physiol.

[9] Axtell MJ, Jan C, Rajagopalan R, Bartel DP (2006). A two-hit trigger for siRNA biogenesis in plants. Cell.

[10] Axtell MJ, Westholm JO, Lai EC (2011). Vive la difference: biogenesis and evolution of microRNAs in plants and animals. Genome Biol.

[11] Gebelin V (2012). Identification of novel microRNAs in Hevea brasiliensis and computational prediction of their targets. BMC Plant Biol.

[12] An W (2015). MicroRNA and mRNA expression profiling analysis revealed the regulation of plant height in Gossypium hirsutum. BMC Genomics.

[13] Addo-Quaye C, Eshoo TW, Bartel DP, Axtell MJ (2008). Endogenous siRNA and miRNA targets identified by sequencing of the Arabidopsis degradome. Curr Biol.

[14] Pappas Mde C, Pappas GJ, Grattapaglia D (2015). Genome-wide discovery and validation of Eucalyptus small RNAs reveals variable patterns of conservation and diversity across species of Myrtaceae. BMC Genomics.

[15] Chen M, Bao H, Wu Q, Wang Y (2015). Transcriptome-Wide Identification of miRNA Targets under Nitrogen Deficiency in Populus tomentosa Using Degradome Sequencing. International journal of molecular sciences.

[16] de Faÿ, E. & Jacob, J. L. In *Physiology of rubber tree latex* (eds J. d’Auzac, J. L. Jacob, & H. Chrestin) 4–14 (CRC Press, 1989).

[17] d’Auzac, J. *et al*. In *Recent research developments in plant physiology* Vol. 1 (ed. Pandalai S. G.) 273–332 (Trivandum: Research Singpost, 1997).

[18] Chrestin H, Bangratz J, d’Auzac J, Jacob J (1984). Role of the lutoidic tonoplast in the senescence and degeneration of the laticifers of Hevea brasiliensis. Zeitschrift für Pflanzenphysiologie.

[19] Putranto RA (2015). Involvement of Ethylene in the Latex Metabolism and Tapping Panel Dryness of Hevea brasiliensis. International journal of molecular sciences.

[20] Wititsuwannakul R, Pasitkul P, Jewtragoon P, Wititsuwannakul D (2008). Hevea latex lectin binding protein in C-serum as an anti-latex coagulating factor and its role in a proposed new model for latex coagulation. Phytochemistry.

[21] Okoma KM, Dian K, Obouayeba S, Elabo AAE, N’guetta ASP (2011). Seasonal variation of tapping panel dryness expression in rubber tree *Hevea brasiliensis* muell.arg. in Côte d’Ivoire. Agriculture and Biology Journal of North America.

[22] Zhang Y, Leclercq J, Montoro P (2016). Reactive oxygen species in Hevea brasiliensis latex and relevance to Tapping Panel Dryness. Tree Physiol.

[23] Chao J, Chen Y, Wu S, Tian WM (2015). Comparative transcriptome analysis of latex from rubber tree clone CATAS8-79 and PR107 reveals new cues for the regulation of latex regeneration and duration of latex flow. BMC Plant Biol.

[24] Li H-L (2016). Comparative Transcriptome Analysis of Latex Reveals Molecular Mechanisms Underlying Increased Rubber Yield in Hevea brasiliensis Self-Rooting Juvenile Clones. Frontiers Plant Science.

[25] Tang C (2016). The rubber tree genome reveals new insights into rubber production and species adaptation. Nature Plants.

[26] Montoro P (2018). Transcriptome analysis in Hevea brasiliensis latex revealed changes in hormone signalling pathways during ethephon stimulation and consequent Tapping Panel Dryness. Sci Rep.

[27] Wei F (2015). Transcriptome sequencing and comparative analysis reveal long-term flowing mechanisms in Hevea brasiliensis latex. Gene.

[28] Li D (2015). Next-generation sequencing, assembly, and comparative analyses of the latex transcriptomes from two elite *Hevea brasiliensis* varieties. Tree Genetics & Genomes.

[29] Gébelin V, Leclercq J, Hu S, Tang C, Montoro P (2013). Regulation of MIR genes in response to abiotic stress in Hevea brasiliensis. International journal of molecular sciences.

[30] Gébelin, V. *et al*. The small RNA profile in latex from Hevea brasiliensis trees is affected by tapping panel dryness. *Tree Physiology*, 10.1093/treephys/tpt076 (2013). 10.1093/treephys/tpt076 24218245

[31] Kanjanawattanawong S (2014). Characterization of rubber tree microRNA in phytohormone response using large genomic DNA libraries, promoter sequence and gene expression analysis. Molecular genetics and genomics.

[32] Lertpanyasampatha M (2012). Genome-wide analysis of microRNAs in rubber tree (Hevea brasiliensis L.) using high-throughput sequencing. Planta.

[33] Lertpanyasampatha M, Viboonjun U, Kongsawadworakul P, Chrestin H, Narangajavana J (2014). Differential expression of microRNAs and their targets reveals a possible dual role in physiological bark disorder in rubber tree. Journal of Plant Physiology.

[34] Pramoolkit P (2014). Involvement of ethylene-responsive microRNAs and their targets in increased latex yield in the rubber tree in response to ethylene treatment. Plant Physiol Biochem.

[35] Chrestin, H. In *Physiology of Rubber* Tree Latex (eds d’Auzac, J., Jacob, J. L. & Chrestin, C.) 431–441 (CRC Press, Inc., 1989).

[36] Bulley S, Laing W (2016). The regulation of ascorbate biosynthesis. Curr Opin Plant Biol.

[37] Conklin PL (1999). Genetic evidence for the role of GDP-mannose in plant ascorbic acid (vitamin C) biosynthesis. Proc Natl Acad Sci USA.

[38] Dunphy PJ, Whittle KJ, Pennock JF, Morton RA (1965). Identification and estimation of tocotrienols in Hevea latex. Nature.

[39] Yacob AR, Bakar NAA, Said N (2012). Vitamin E Isomers from Latex Timber Clone Rubber Tree Characterized by Ultra Violet and High Performance Liquid Chromatography. APCBEE Procedia.

[40] Mellidou I, Chagne D, Laing WA, Keulemans J, Davey MW (2012). Allelic variation in paralogs of GDP-L-galactose phosphorylase is a major determinant of vitamin C concentrations in apple fruit. Plant Physiol.

[41] Stevens R (2008). Tomato fruit ascorbic acid content is linked with monodehydroascorbate reductase activity and tolerance to chilling stress. Plant Cell Environ.

[42] Sauvage C (2014). Genome-Wide Association in Tomato Reveals 44 Candidate Loci for Fruit Metabolic Traits. Plant Physiol.

[43] Jo Y, Hyun TK (2011). Genome-wide identification of antioxidant component biosynthetic enzymes: comprehensive analysis of ascorbic acid and tocochromanols biosynthetic genes in rice. Computational biology and chemistry.

[44] Sohpal VK, Dey A, Singh A (2010). MEGA biocentric software for sequence and phylogenetic analysis: a review. International journal of bioinformatics research and applications.

[45] Xiong E, Zheng C, Wu X, Wang W (2016). Protein subcellular location: The gap between prediction and experimentation. Plant Molecular Biology Reporter.

[46] German MA, Luo S, Schroth G, Meyers BC, Green PJ (2009). Construction of Parallel Analysis of RNA Ends (PARE) libraries for the study of cleaved miRNA targets and the RNA degradome. Nature protocols.

[47] Axtell MJ, Meyers BC (2018). Revisiting Criteria for Plant MicroRNA Annotation in the Era of Big Data. Plant Cell.

