# Supplementary material for: Genome-wide analysis in Hevea brasiliensis laticifers revealed species-specific post-transcriptional regulations of several redox-related genes
Source: Sci Rep. 2019 Apr 5;9:5701. doi: 10.1038/s41598-019-42197-8 (PMC6450977; doi:10.1038/s41598-019-42197-8)
Supplement: Supplementary file 1 — Supplementary figures [file 41598_2019_42197_MOESM1_ESM.pdf]

Genome-wide analysis in *Hevea brasiliensis* laticifers revealed species-specific post-transcriptional regulations of several redox-related genes

Yi Zhang[1,2], Julie Leclercq[1,2], Shuangyang Wu[1,2,3,4], Enrique Ortega-Abboud[1,2], Stéphanie Pointet[1,2], Chaorong Tang[5], Songnian Hu[3], Pascal Montoro[1,2*]

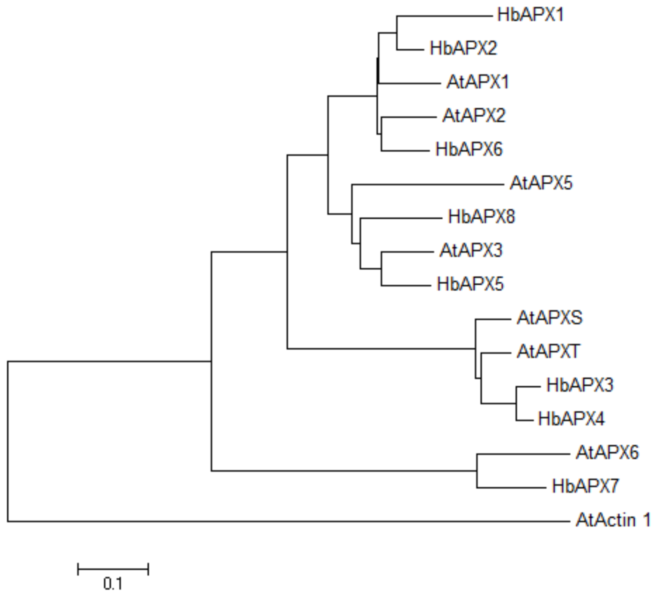

Supplementary Figure 1. Phylogenetic tree of APX (ascorbate peroxidase). *Hevea* and *Arabidopsis* deduced amino acid sequences were aligned using Muscle via Mega 6. The phylogenetic trees were generated in Mega 6 after alignment.

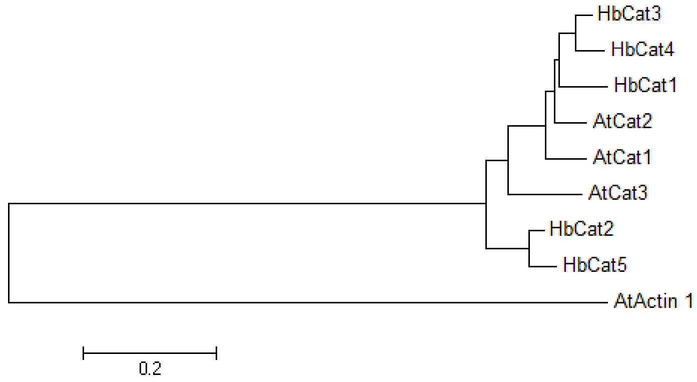

Supplementary Figure 2. Phylogenetic tree of Cat (catalase). *Hevea* and *Arabidopsis* deduced amino acid sequences were aligned using Muscle via Mega 6. The phylogenetic trees were generated in Mega 6 after alignment.

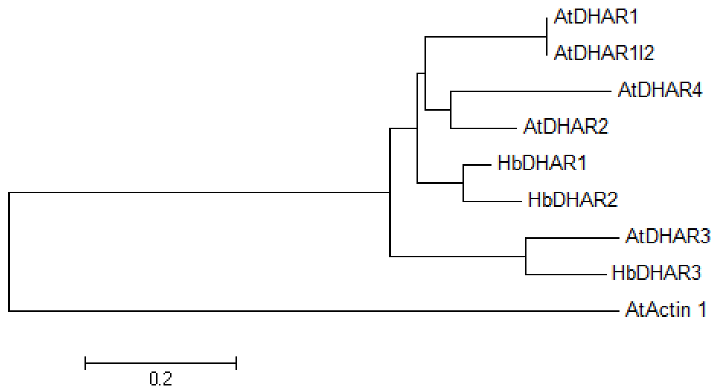

Supplementary Figure 3. Phylogenetic tree of DHAR (dehydroascorbate reductase). *Hevea* and *Arabidopsis* deduced amino acid sequences were aligned using Muscle via Mega 6. The phylogenetic trees were generated in Mega 6 after alignment.

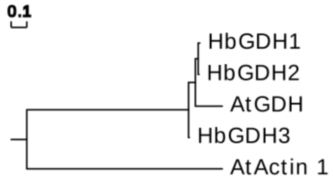

Supplementary Figure 4. Phylogenetic tree of GDH (L-galactose dehydrogenase). *Hevea* and *Arabidopsis* deduced amino acid sequences were aligned using Muscle via Mega 6. The phylogenetic trees were generated in Mega 6 after alignment.

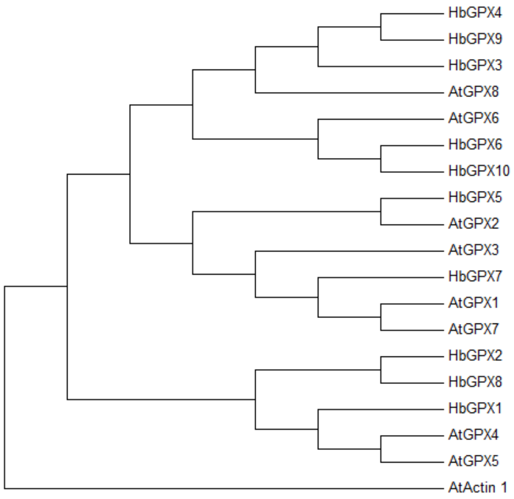

Supplementary Figure 5. Phylogenetic tree of GPX (glutathione peroxidase). *Hevea* and *Arabidopsis* deduced amino acid sequences were aligned using Muscle via Mega 6. The phylogenetic trees were generated in Mega 6 after alignment.

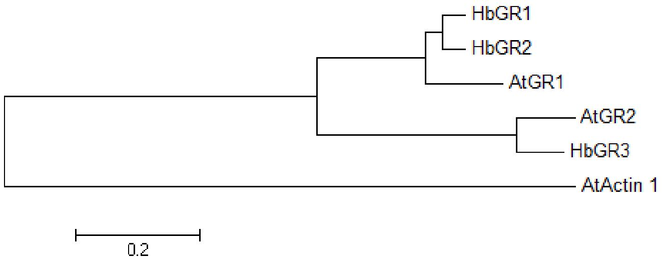

Supplementary Figure 6. Phylogenetic tree of GR (glutathione reductase). *Hevea* and *Arabidopsis* deduced amino acid sequences were aligned using Muscle via Mega 6. The phylogenetic trees were generated in Mega 6 after alignment.

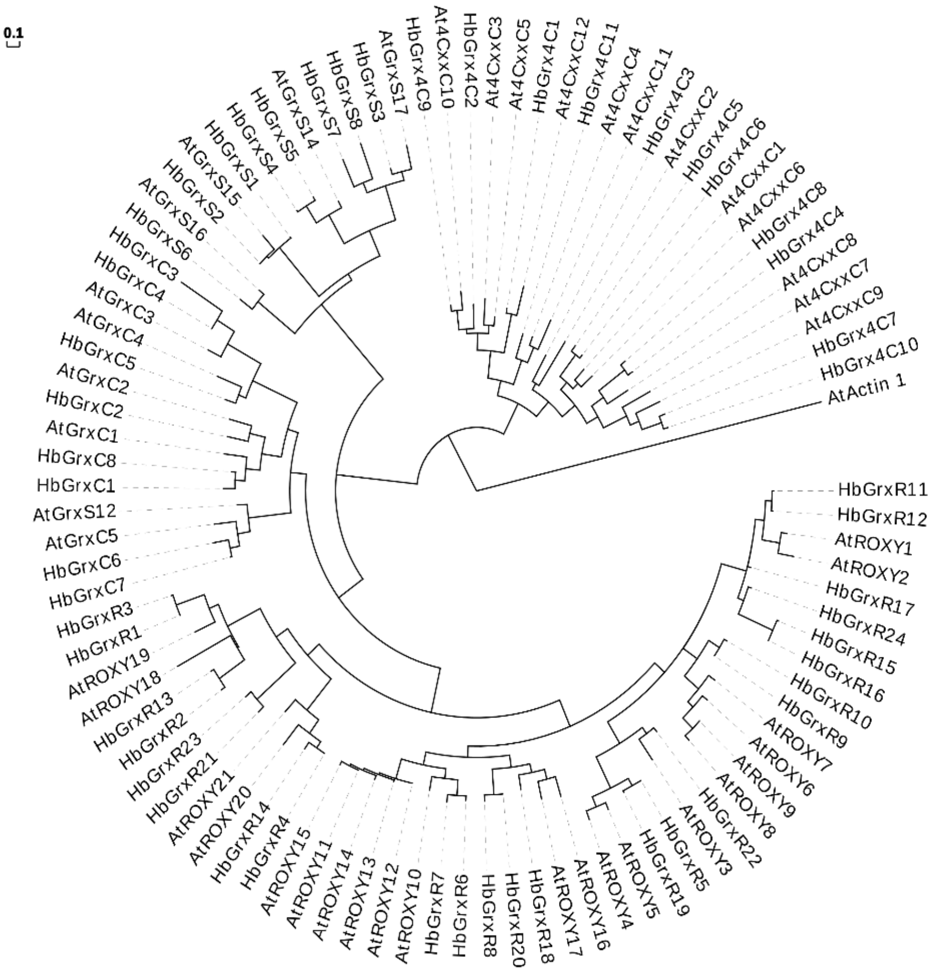

Supplementary Figure 7. Phylogenetic tree of Grx (glutaredoxin). *Hevea* and *Arabidopsis* deduced amino acid sequences were aligned using Muscle via Mega 6. The phylogenetic trees were generated in Mega 6 after alignment.

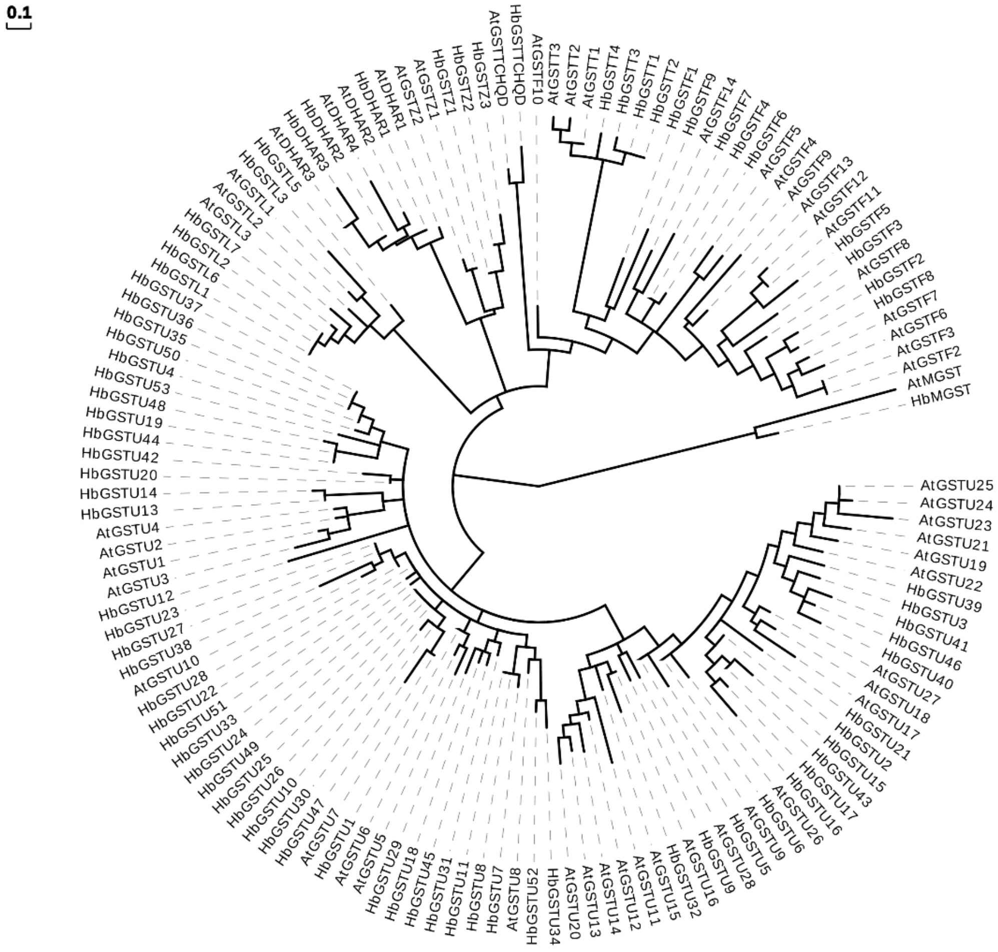

Supplementary Figure 8. Phylogenetic tree of GST (glutathione S-transferase). *Hevea* and *Arabidopsis* deduced amino acid sequences were aligned using Muscle via Mega 6. The phylogenetic trees were generated in Mega 6 after alignment.

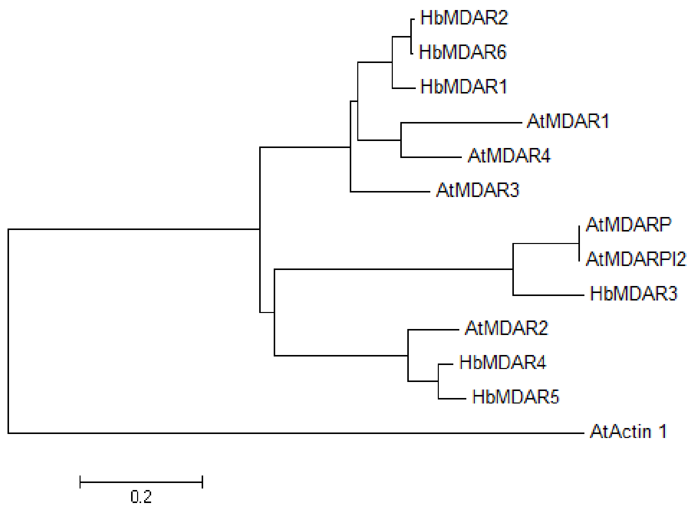

Supplementary Figure 9. Phylogenetic tree of MDHAR (Monodehydroascorbate reductase). *Hevea* and *Arabidopsis* deduced amino acid sequences were aligned using Muscle via Mega 6. The phylogenetic trees were generated in Mega 6 after alignment.

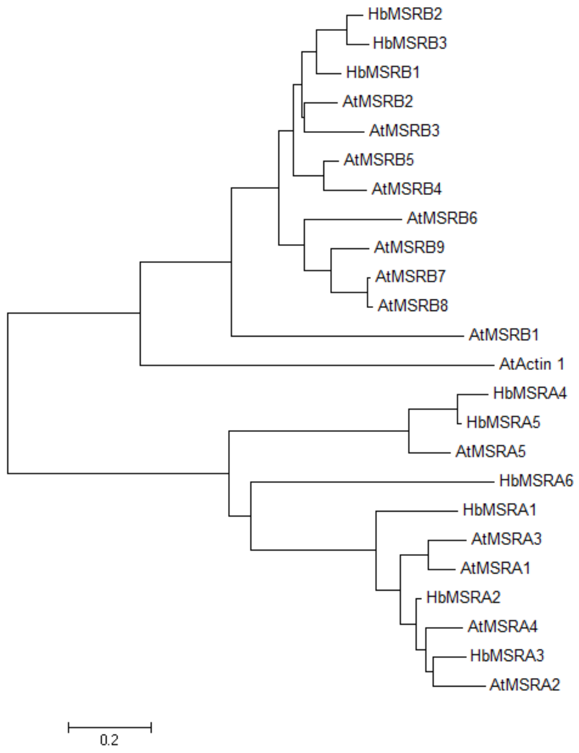

Supplementary Figure 10. Phylogenetic tree of MSR (Methionine sulfoxide reductase). *Hevea* and *Arabidopsis* deduced amino acid sequences were aligned using Muscle via Mega 6. The phylogenetic trees were generated in Mega 6 after alignment.

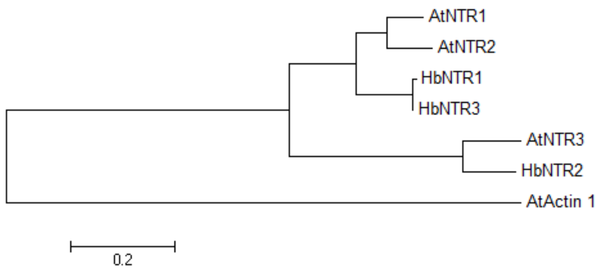

Supplementary Figure 11. Phylogenetic tree of NTR (NADPH-dependent thioredoxin reductase). *Hevea* and *Arabidopsis* deduced amino acid sequences were aligned using Muscle via Mega 6. The phylogenetic trees were generated in Mega 6 after alignment.

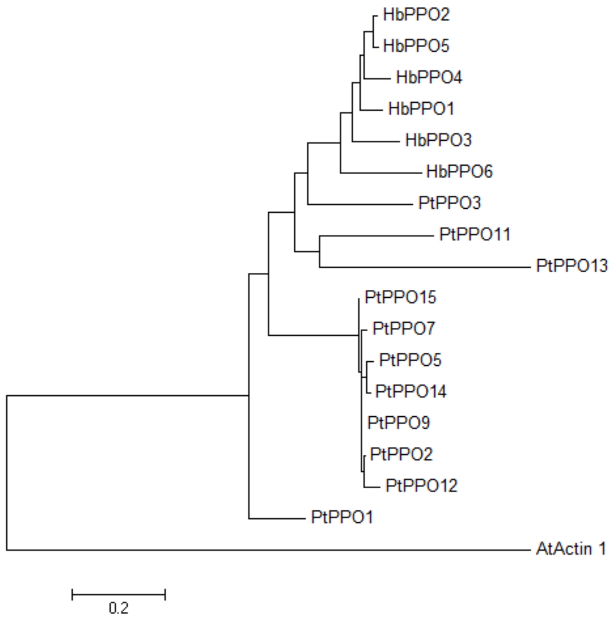

Supplementary Figure 12. Phylogenetic tree of PPO (polyphenol oxidase). *Hevea* and *Populus* deduced amino acid sequences were aligned using Muscle via Mega 6. The phylogenetic trees were generated in Mega 6 after alignment.

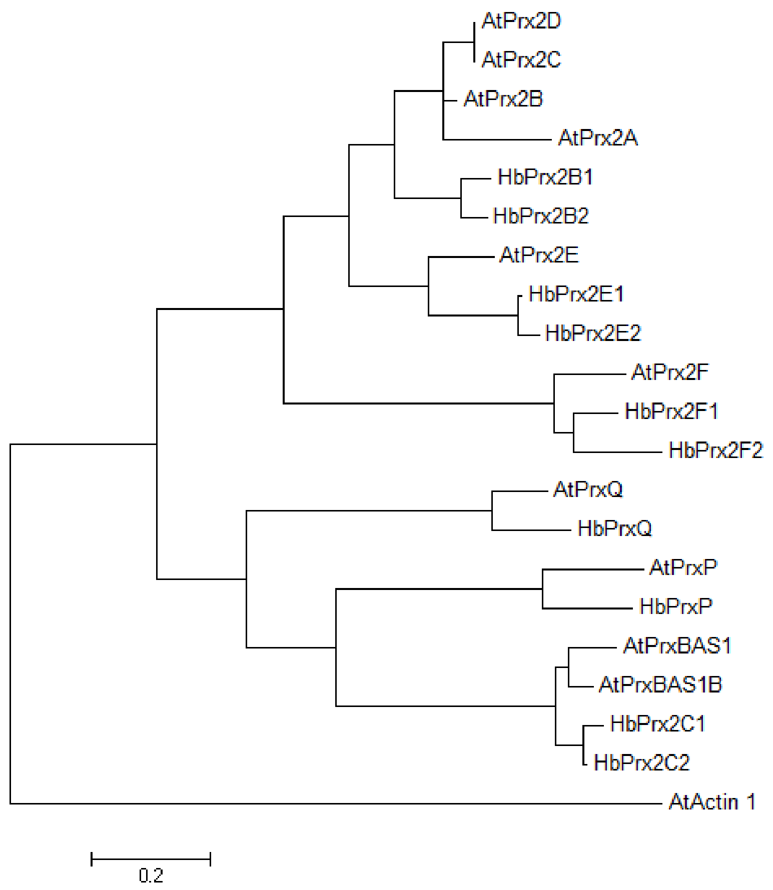

Supplementary Figure 13. Phylogenetic tree of Prx (polyphenol oxidase). *Hevea* and *Arabidopsis* deduced amino acid sequences were aligned using Muscle via Mega 6. The phylogenetic trees were generated in Mega 6 after alignment.

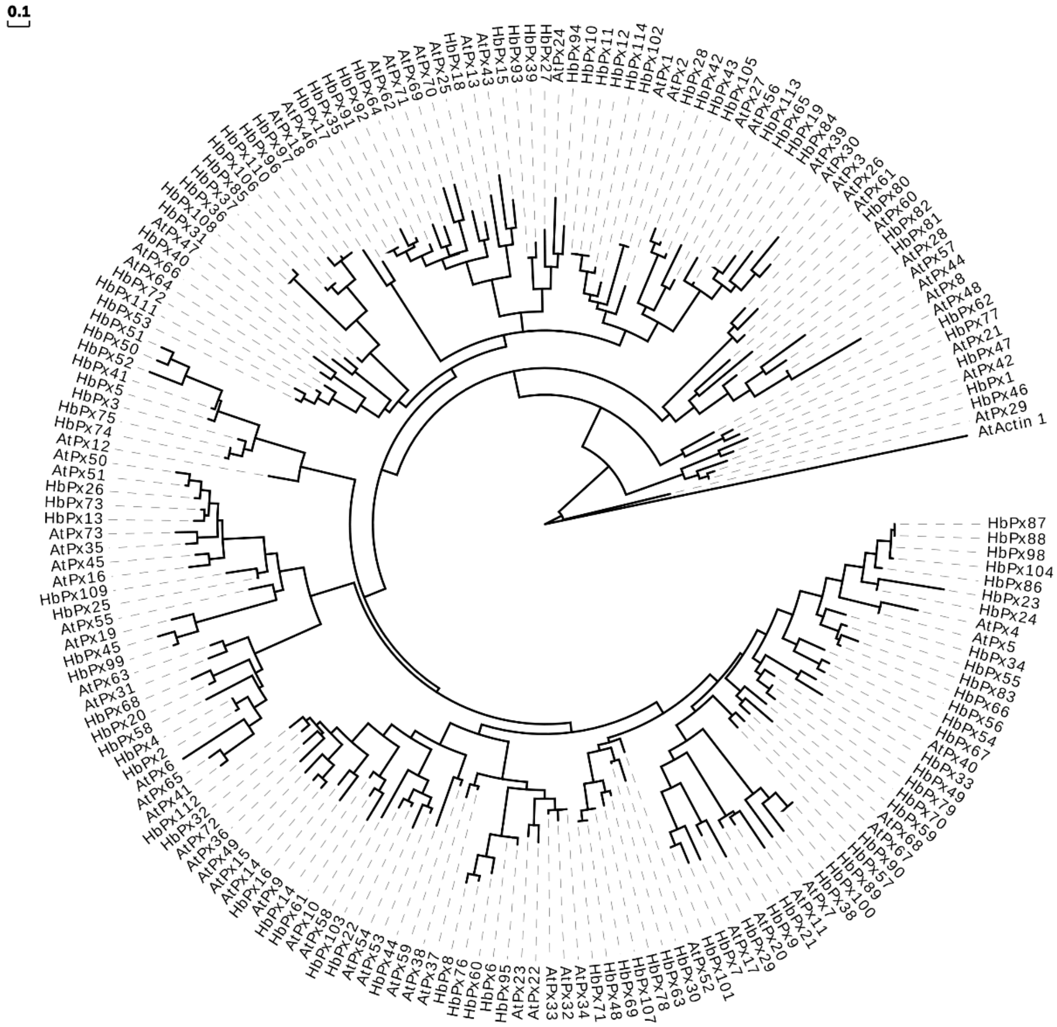

Supplementary Figure 14. Phylogenetic tree of Px (peroxidase). *Hevea* and *Arabidopsis* deduced amino acid sequences were aligned using Muscle via Mega 6. The phylogenetic trees were generated in Mega 6 after alignment.

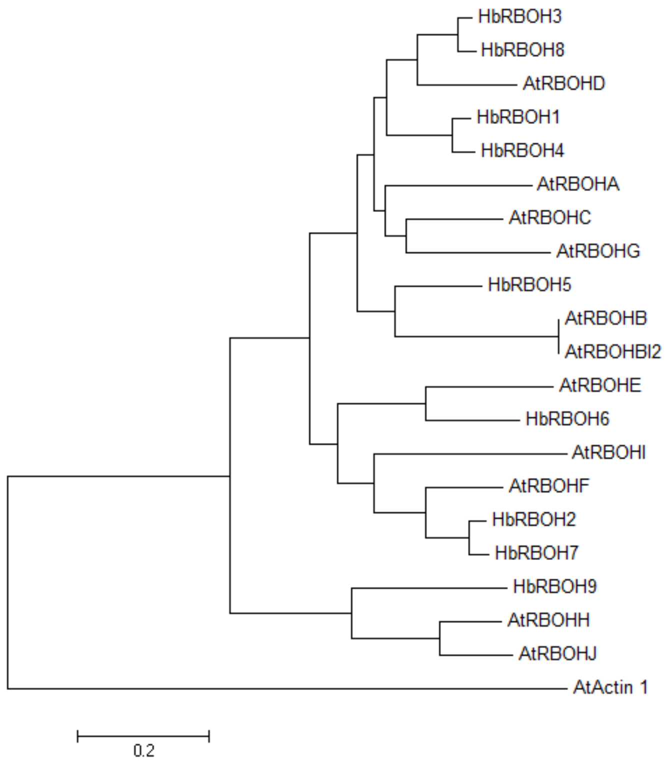

Supplementary Figure 15. Phylogenetic tree of RBOH (Respiratory burst oxidase homolog). *Hevea* and *Arabidopsis* deduced amino acid sequences were aligned using Muscle via Mega 6. The phylogenetic trees were generated in Mega 6 after alignment.

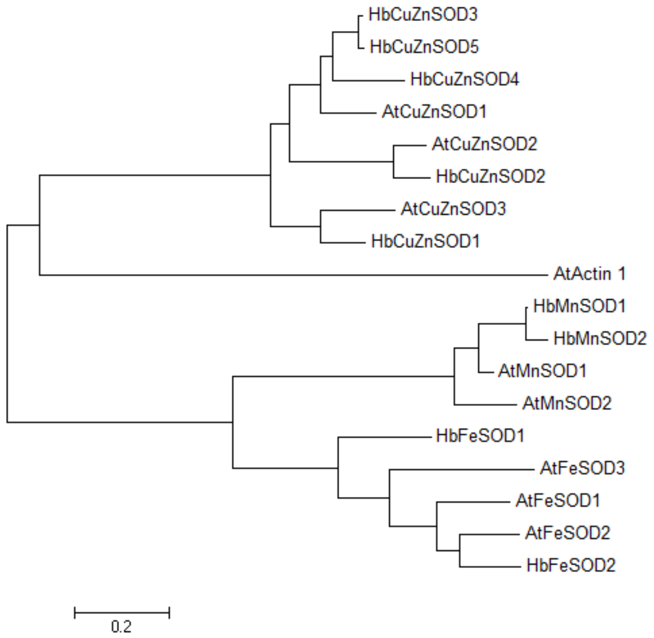

Supplementary Figure 16. Phylogenetic tree of SOD (superoxide dismutase). *Hevea* and *Arabidopsis* deduced amino acid sequences were aligned using Muscle via Mega 6. The phylogenetic trees were generated in Mega 6 after alignment.

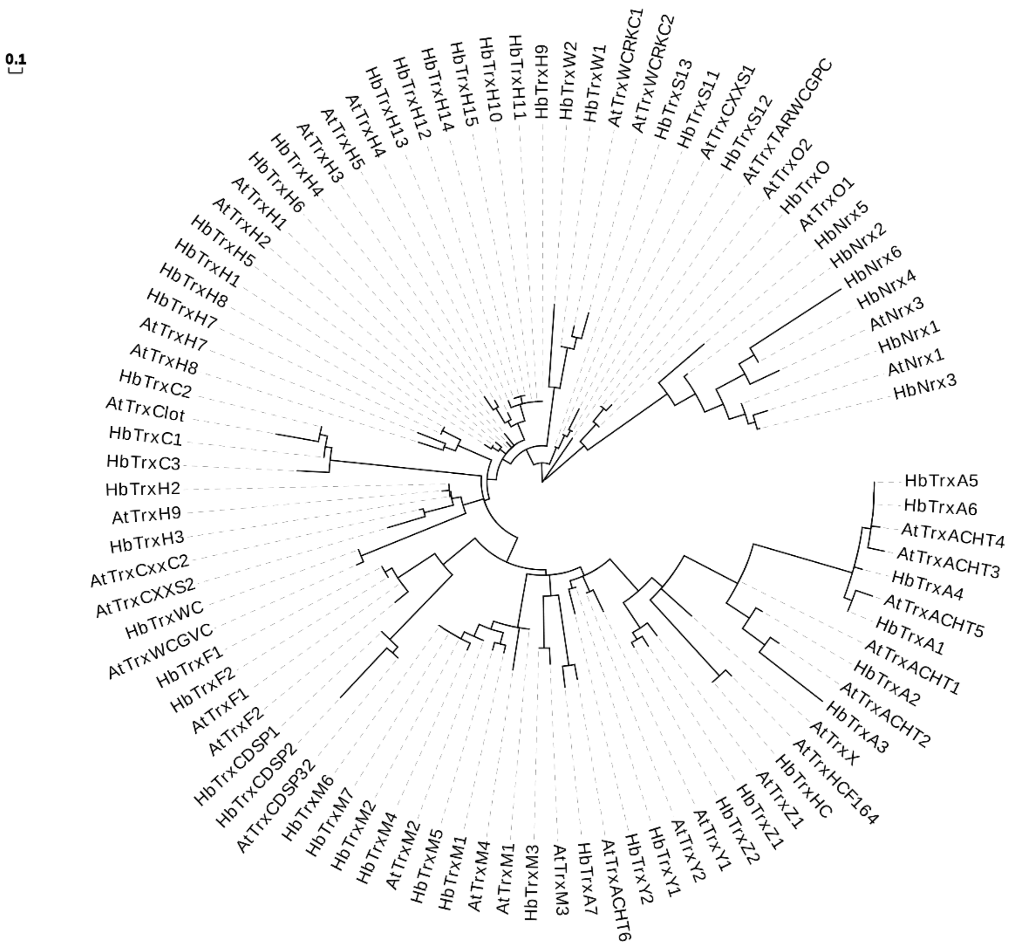

Supplementary Figure 17. Phylogenetic tree of Trx (thioredoxin). *Hevea* and *Arabidopsis* deduced amino acid sequences were aligned using Muscle via Mega 6. The phylogenetic trees were generated in Mega 6 after alignment.
